# Human-driven global nutrient imbalances increase risks to health

**DOI:** 10.1016/j.eehl.2023.08.003

**Published:** 2023-08-24

**Authors:** Josep Penuelas, Jordi Sardans

**Affiliations:** a CSIC, Global Ecology Unit CREAF-CSIC-UAB, 08193 Bellaterra, Barcelona, Catalonia, Spain; b CREAF, 08193 Cerdanyola del Vallès, Barcelona, Catalonia, Spain

**Keywords:** Nitrogen, Phosphorus, N:P ratio, Non-infectious diseases, Infectious diseases, Medical treatments

## Abstract

Human-induced inputs of nitrogen (N) and phosphorus (P) into the biosphere have reached unprecedented levels, particularly N, leading to an escalating global anthropogenic N:P ratio. This ratio has emerged as a significant driver of environmental change, impacting organisms, ecosystems, and global food security. However, the implications of this ratio for human health have been largely overlooked and remain uncertain. This article aims to fill this knowledge gap by exploring the potential effects of N:P ratios on both non-infectious and infectious diseases. Preliminary data emphasize the importance of investigating the influence of N:P ratios on human health, suggesting a potential role in the rise of non-infectious diseases, such as cancer, as well as the proliferation of infectious diseases, including Zika and malaria. These findings highlight the urgent need for increased attention from the scientific community and policymakers regarding the complex impacts of the human-induced biospheric N:P ratio. It is crucial to investigate and understand the underlying mechanisms and drivers behind these effects. Furthermore, there is significant potential for improving human health through the manipulation of N:P ratios and the availability of N and P. This applies not only to medical treatments but also to innovative fertilizer management strategies. These avenues present promising opportunities to address the challenges associated with human health in an ever-changing world.

## Highlights

• Unprecedented N:P ratio impacts environment, organisms, food security.
• N:P ratios may affect non-infectious and infectious diseases.
• Urgent need to study N:P ratio effects on human health.
• Potential for improving health through N:P manipulation.
• N:P ratios offer opportunities for addressing health challenges.

## Graphical abstract

**Figure:**
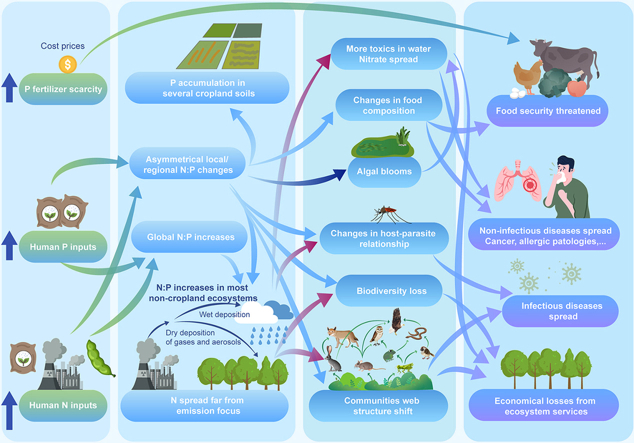

## 1 Human impacts on nitrogen and phosphorus cycles

In recent decades, human activities have had a profound impact on the nitrogen (N) and phosphorus (P) cycles, leading to unprecedented releases of N into the environment and resulting in a nutrient imbalance between N and P in the biosphere [1]. Anthropogenic inputs of reactive N to the biosphere, driven by fossil fuel combustion and fertilizer use, have increased significantly, contributing to annual N releases of 25–33 Tg N and >150 Tg N, respectively. Additionally, leguminous crops and rice fix approximately 90 Tg N per year from atmospheric N_2_. Some regions have also experienced N eutrophication [2]. On the other hand, anthropogenic releases of P have remained relatively stable and lower compared to N, primarily originating from fertilizer use (22–26 Tg P per year) and animal feed. However, these human-induced P fluxes, although comparable to natural global fluxes [3], have been shown to cause regional soil and water eutrophication, influencing the global biogeological P cycle.

The difference in the anthropogenic release of reactive N and P has led to an overall increase in the global mean N:P ratio, which now stands at approximately 30 on a molar basis for anthropogenic inputs, while values for principal ecosystem components range from 16 (ocean water, plankton) to 22 (soils). However, at local-regional scales, the imbalance in N and P inputs may be even greater due to uneven distribution and differences in environmental mobility. P tends to have low water solubility and volatilization, often adsorbing and precipitating in the soil as salt minerals or being buried in sediments near emission sources. In contrast, N is more water-soluble and volatile, spreading over larger distances from its sources [4].

P enrichment is common in agricultural soils, as it has low solubility and a tendency to precipitate and become immobilized. This has led to declines in N:P ratios in several parts of the world [4,5], particularly in regions with high livestock density where manure waste exhibits very low N:P ratios [6]. While most continental and coastal areas have experienced increased N:P ratios due to N spreading capacity, cropland soils and adjacent habitats receiving untreated or diffuse wastes and leachates have witnessed decreased N:P ratios.

The N:P ratio plays a crucial role in determining community structure and function, especially when concentrations of both N and P are not limiting. Plankton’s protein synthesis rate, for instance, is dependent on the amount of P-rich RNA produced, leading to a negative correlation between cellular N/P ratio and protein synthesis rate, resulting in faster growth rates of plankton with lower N/P ratios [7]. This negative correlation has ecological consequences for the structure and functioning of ecosystems, as observed in various ecosystem types [4].

In addition to N and P imbalances, human activities have also caused imbalances among other elements, such as carbon (C), N, iron, zinc, calcium, and potassium, in plant tissues [8,9]. Increasing atmospheric CO_2_ concentrations are likely drivers of increased C concentrations in plants, consequently reducing the concentrations of these other elements [8,9].

**Fig. 1:**
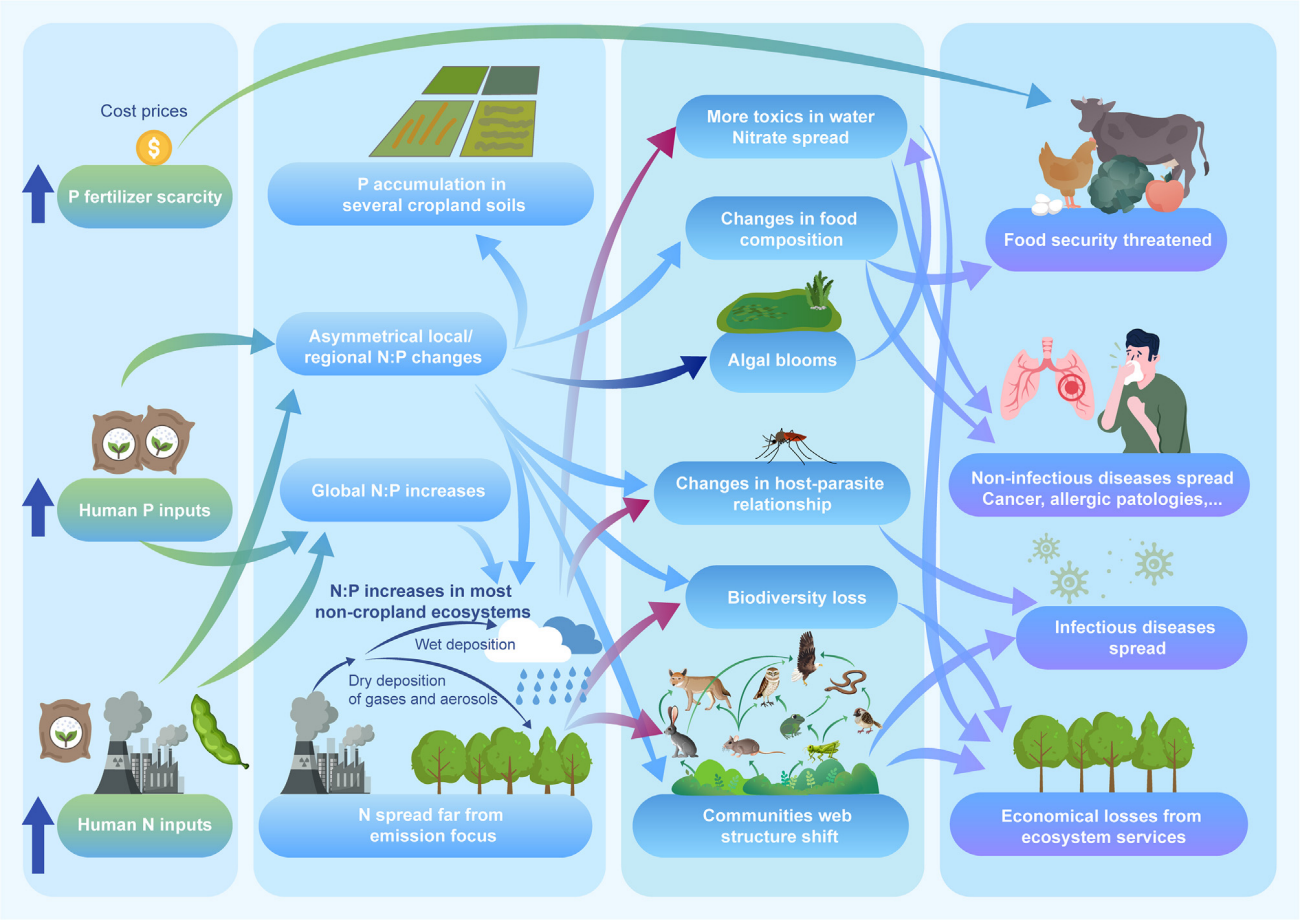
Schematic diagram of the impact of N:P on human food security and health.

While the environmental impact of human-driven N and P inputs on managed and semi-natural ecosystems and food security is well-documented [[1], [2], [3], [4]], their direct effects on human health are less understood. This article aims to review current research on the potential impacts of N:P ratios on human health (Fig. 1), shedding light on this important yet understudied aspect.

## 2 Impact of nutrient imbalances on human health

### 2.1 Growth

Humans, like other species, are profoundly influenced by elemental and stoichiometric changes in their environment. The impact of anthropogenic shifts in the input of N and P into the biosphere, and the resulting changes in N:P ratios, have cascading effects on the human food chain [10,11]. In the past, the excessive use of N fertilizer in economically advanced countries has led to food overproduction, while low levels of fertilizer use in economically developing countries have helped prevent malnutrition [12]. These disparities in fertilizer use have contributed to variations in human growth and development. Notably, variations in per capita intake of N and P, as well as the associated balance in the N:P ratio, correlate with differences in the average height of men born in high-income countries. This correlation is even more significant than socioeconomic or sanitary variables such as GDP, the human development index, and birth weight, as indicated by integrated analyses of data from FAO (2006), OECD (2022), and WHO [10].

### 2.2 Non-infectious diseases

#### 2.2.1 Nitrogen immunological and oncological effects

The positive effects of increased N and P intake on human growth and development are accompanied by potential risks to the triggers of non-infectious diseases. For instance, the global annual N fertilization of wheat has significantly increased from 9.8 to 93.8 kg/ha between 1961 and 2010 [13], reflecting the overall rise in N fertilizers across different types of farmland (11.3–107.6 Tg) [14]. This escalation in N fertilization has raised concerns about the potential increase in the risk of health disorders, including coeliac disease, due to the elevated content of allergenic proteins [15], such as gluten [16,17], particularly the gliadin fraction [[18], [19], [20]], which is known to trigger and sustain this disease [[21], [22], [23]]. Notably, a higher availability of N has been associated with increased expression of gliadin genes [20].

Anthropogenic increases in N, particularly in its active/reactive forms such as nitrate (NO_3_^−^) and ammonium, and exposure to atmospheric nitrogen dioxide in occupational and domestic settings, can have adverse effects on human health. However, the pervasive presence of environmental pollutants derived from reactive N complicates the interdisciplinary assessment of their harmful effects on human health [24].

The increasing concentrations of nitrates in groundwater resulting from surface leaching of fertilizers, as well as industrial, animal, and human waste, pose health risks when consumed in drinking water, especially for children under five years old [25]. For example, the leaching of livestock waste from cropland has led to high levels of water pollution in aquifers in Europe, China, India, Reunion Island, and various regions of the USA, Canada, and Australia [[26], [27], [28], [29], [30], [31], [32], [33]]. Nitrates and nitrites are also used *in processed meats* as additives in meat curing [34] and their intake has been related to breast and prostate cancer risk in humans [35].

#### 2.2.2 Phosphate and cancer

High dietary consumption of phosphate (PO_4_) has been found to stimulate the growth of lung and skin tumours in experimental animals, potentially due to increased growth-promoting cell signaling, neovascularization, and chromosome instability in the presence of excessive phosphate [36].

Tumour cells are known to exhibit higher expression levels of phosphate cotransporters and accumulate larger quantities of inorganic phosphate compared to healthy cells [36,37]. For example, lung and colon tumours have been found to contain approximately double the amount of P and RNA concentration compared to healthy tissue, resulting in lower N:P ratios [38,39]. Elser et al. [[38], [39], [40][38], [39], [40]] analyzed the elemental composition of normal and tumour tissues from the lung, colon, liver, and kidney. Consistent with the Growth Rate Hypothesis (GRH), they observed that lung and colon tumours exhibited significantly higher P and RNA concentrations (as a fraction of dry weight) and lower N:P ratios compared to their respective normal tissue. However, they did not find significant differences in %P and %RNA between malignant and normal tissues in the liver and kidney tumour tissues. Moreover, the P present in RNA accounted for a significantly larger fraction of the total biomass P in malignant tissues than in normal tissues. These findings support the GRH, which suggests that under conditions where neither N nor P is limiting, a low N:P ratio is associated with a high growth rate, as P-rich RNA becomes the limiting factor for synthesizing amino acids and building proteins.

Considering the growing evidence linking phosphate and N:P ratios to certain cancer incidences in humans, it is crucial to consider the human-driven impacts on global P and N cycles [3,41] and their potential consequences on P concentrations and N:P ratios in food. Accumulation of P in some cropland soils has been reported, leading to increased P uptake by crops and potentially influencing food composition by elevating P concentrations [[42], [43], [44]]. Although studies have shown high levels of P uptake in both crops and non-crop plants under conditions of high soil P concentrations, the global-scale link between P accumulation in crop soils, P concentrations in food, and the resulting implications for human health remains unknown. Further research is needed to explore the effects of increased dietary P intake, as excessive P intake has been inconsistently linked to health problems such as bone health, cancer risk, and heart failure associated with the augmented use of P additives in foods [[45], [46], [47]].

Several reports have highlighted the links between cancer incidence and phosphate levels in the human body. Chromosome instability, a characteristic of cancer cells, has been positively associated with high phosphate concentrations. For example, hyperphosphatemia has been shown to increase the risk of chromosome aberrations in the parathyroid glands of patients with hyperparathyroidism [48], and high levels of dietary phosphate have been linked to increases in lymphocyte genotoxicity [49]. A 24-year monitoring study as part of the Health Professionals Follow-Up Study revealed that high levels of dietary phosphate are independently associated with lethal and high-grade prostate cancer [50]. Milk and dairy products contribute the highest percentage of phosphate intake in the American diet [51]. Newmark and Heaney proposed that phosphate from dairy products is a risk factor correlated with prostate cancer in epidemiological studies of American male physicians and Swedish males [52]. In Swedish women, cancer (all types) mortality increased by 44% (hazard ratio 1.44; 95% confidence interval 1.23–1.69) when consuming three glasses of milk per day compared to one glass [53]. In a clinical study of 9,686 adults aged 20–80, with analyses adjusted for demographics, cardiovascular risk factors, kidney function, and energy intake, higher phosphorus intake was associated with higher all-cause mortality in individuals, with this association being particularly significant among individuals with a daily phosphorus intake exceeding 1,400 mg/d [54].

These findings emphasize the complex relationship between phosphate levels, dietary intake, and cancer incidence. Tumour cells exhibit altered phosphate accumulation and metabolism, which can contribute to their growth and proliferation. Furthermore, the accumulation of phosphorus in certain cropland soils and its subsequent uptake by crops raise concerns about elevated phosphorus concentrations in food. However, the direct implications of these changes on human health are still not fully understood.

Further research is needed to explore the specific mechanisms underlying the association between phosphate levels and cancer development. Additionally, investigating the global-scale link between phosphorus accumulation in crop soils, phosphorus concentrations in food, and their impact on human health is crucial. Understanding the effects of excessive dietary phosphorus intake and the use of phosphorus additives in food production will help to elucidate potential health risks and guide strategies for maintaining a balanced and healthy diet.

In conclusion, medical studies have underscored the role of phosphate toxicity as a pathophysiological determinant in cancer cell growth, with tumour cells exhibiting higher expression levels of phosphate cotransporters and storing larger amounts of inorganic phosphate compared to normal cells. Disruption of phosphate homeostasis has been associated with the development of various human tumours [36].

#### 2.2.3 Therapies for cancer

Chemotherapy typically results in a significant reduction in tumour populations, often by a factor of a thousand, before resistance emerges. However, complete eradication of the tumour population is essential to prevent tumour recurrence, as the extracellular conditions following chemotherapy can stimulate the proliferation of surviving tumour cells due to the release of substantial amounts of phosphate and other cellular material upon tumour cell death [55]. Therefore, integrating stoichiometric theory into cancer treatment strategies may enhance the efficacy of chemotherapy. For example, post-chemotherapy dialysis, which selectively removes circulating phosphate from the blood plasma, could be considered to minimize the proliferation of surviving tumour cells and facilitate improved immune system management [40].

However, it is not only phosphate itself that plays a critical role; the N:P ratios may also be crucial in enhancing the effectiveness of cancer therapies. One promising approach in combating cancer growth involves targeting the p53 tumour suppressor gene. The successful application of this therapy relies on the specific delivery of the p53 gene to cancer cells. The protein carriers involved in the process possess structures—such as morphology, size, and surface charges—that are highly influenced by the N:P ratio (the ratio of nitrogen to phosphate groups). It has been observed that the N:P ratio significantly impacts the efficiency of gene transfection, a process that involves introducing nucleic acids into eukaryotic cells using nonviral methods. Thus, the N:P ratio can be precisely adjusted to achieve desired levels of protein expression and apoptosis. The notable advantage of this proposed system lies in the use of N:P ratios as a customizable parameter that not only modulates vector properties but also controls the extent of plasmid DNA delivery, protein production, and, consequently, the efficacy of p53-mediated cancer therapy [56,57]. Therefore, manipulating N:P ratios through dietary interventions or other strategies represents an additional tool that warrants investigation in the fight against certain types of human non-infectious diseases.

### 2.3 Infectious diseases

There is emerging evidence that gaining a better understanding of the role of environmental stoichiometry in the structure, organization, and function of human microbial communities can contribute to the management and control of disease vectors. Analysis of stoichioproteomics in different body-site locations has revealed variations in the N content of microbial proteins, with skin and nasal proteins, for example, being particularly nitrogen-rich. These varying proportions of bio-elements in proteins from distinct organ tissues are likely linked to the local environments at different human body sites, including atmospheric exposure and food intake rates [58]. Moreover, as atmospheric carbon dioxide levels increase globally due to the burning of fossil fuels, human plant food sources show increased C:N and C:P ratios [59,60], suggesting that humans are altering the elemental stoichiometry that we provide to our symbiotic gut bacteria. This can have impacts on the species structure of gut microflora, which requires further analysis.

Nutrient stoichiometry, such as N:P ratios, may influence traits, growth, and the geographical distribution of human parasites, thereby impacting the incidence and severity of vector-borne diseases. Paseka and Grunberg [61] examined 27 macroparasite taxa from four phyla that infect vertebrate and invertebrate hosts in freshwater ecosystems in New Jersey. They observed that the allometric scaling of phosphorus across species supports the GRH, which predicts that smaller taxa require more phosphorus to support their relatively higher growth rates. Across all species, parasite phosphorus concentrations scaled negatively with body size, while C:P ratios scaled positively. Similar relationships between parasite phosphorus concentrations and body size were observed at the phylum level and within individual species. Nitrogen concentrations and C:N ratios were related to the life-cycle stage, with non-reproductive parasite stages having lower nitrogen concentrations and higher C:N ratios than actively reproducing parasites. Organismal stoichiometry was found to be linked to ecological function, and the wide variation in macroparasite stoichiometry likely generates diverse patterns in host-parasite nutrient dynamics, as well as variable relationships between parasitism and nutrient cycling. This suggests how the N:P availability ratio in the environment (water and soil) can affect parasites and, consequently, the performance of several human infectious diseases.

Aalto et al. [62] reviewed shifts in host-parasite interactions under changing nutrient ratios and observed that the outcome of infection may depend on the overlap in the stoichiometric requirements of the host and the parasite. The authors further hypothesized that environmental nutrient enrichment alters infectivity dynamics, leading to fluctuating selection dynamics in host-parasite coevolution. Shifts in parasite stoichiometry that result in changes in ecosystem function [61] are likely to drive patterns in host-parasite nutrient dynamics, including nutrient cycling, as well as the transmission and prevalence of vectored diseases. These dynamics may depend on overlaps in stoichiometric requirements between hosts and parasites and could potentially impact host-parasite coevolutionary processes [62]. In a long-term chemostat experiment conducted by Larsen et al. [63], the marine cyanobacterium *Synechococcus* was challenged with a lytic phage while manipulating nutrient stoichiometry. The researchers observed alterations in the stability of host-parasite interactions and the underlying mode of coevolution. Nutrient stoichiometry can affect eco-evolutionary feedbacks in ways that may alter the dynamics and functioning of environmental and host-associated microbial communities.

Shifts in environmental N:P ratios are likely to impact globally important human diseases, such as mosquito-vectored malaria, which annually causes an estimated 2 million deaths, with particularly severe effects on children under the age of five [64]. Ecosystem processes operating on various spatio-temporal scales drive the diversity and abundance of aquatic mosquito larvae that may carry malaria-causing protist parasites (*Plasmodium* spp.). For instance, shifts in nutrient stoichiometry can affect aquatic plant communities, which provide protection from mosquito predators and serve as substrate for bacteria consumed by mosquito larvae. These shifts can have consequences for the composition of the mosquito community, including species with varying degrees of vectoring efficiency. Studies conducted in subtropical, P-limited oligotrophic wetlands have demonstrated mechanistic links between nutrient enrichment, wetland vegetation, and vector production. In these wetlands, P-enriched runoff from agricultural land and human settlements has resulted in the replacement of sparse macrophytes (rush) by tall, dense species (cattails), impacting the larval mosquito community [65,66]. Similarly, increased N:P ratios in a western Mediterranean river basin, attributed to improved wastewater treatment, have led to higher macrophyte density, and consequently to increased abundance, and expansion of populations of the black fly (*Simulium erythrocephalum*), a vector of several pathogenic microbes [67,68].

Overlap in parasite-host stoichiometric requirements has been observed, particularly in the case of N:P ratios in a freshwater cyanobacterium host (*Planktothrix rubescens*), which were positively correlated with experimental N:P ratios. These host responses were mirrored in the N:P stoichiometry and the production of toxic microcystins by the fungal parasite *Rhizophydium megarrhizum* (Chytridiomycetes), while zoospore size exhibited a negative association with N:P ratios [69]. Consequently, fungal parasites demonstrate a trade-off between zoospore size and production. As zooplankton graze on chytrid zoospores, changes in parasite production, stoichiometry, and zoospore cell size can have implications for aquatic food web dynamics.

The GRH provides evidence of how parasite–host relationships are influenced by N:P ratios, as the growth rates of hosts with low C:P-ratio diets are positively associated with levels of viral infections [70] and bacterial infections [71]. Although the understanding of this relationship in human pathogenic parasites is limited [72], this association could be of concern for human health in countries where diets are high in P [73]. For example, N:P ratios drive the growth of non-pathogenic bacteria and associated blooms in aquatic systems [7,11], suggesting that shifts in N:P ratios might also influence the growth and proliferation of pathogenic bacteria, such as *Vibrio cholerae*, the causative agent of cholera, and heterotrophic bacteria in aquatic environments [72].

Effects of shifts in environmental stoichiometry on the incidence of vector-borne parasitic diseases in humans may be influenced by their impacts on hosts [74]. This is evident from the effects of aquatic ecosystem stoichiometry on the distribution and population dynamics of larval mosquitoes, which in turn affect adult mosquitoes [75,76]. For instance, mosquito larval hosts of Zika virus exhibit accelerated growth and earlier maturation when provided with an N-rich diet featuring high N:P ratios. This results in a shorter transmission period and reduced risk of infection in humans [77].

Consequently, the relationship between environmental nutrient enrichment and pathogen abundance is intricate, and it varies depending on factors such as microbe type, host/vector density and distribution, pathogen virulence and toxicity, ecosystem conditions, degree of enrichment, and the life cycles of the parasites involved. Such effects may exacerbate the incidence of diseases caused by generalist parasites with direct or simple life cycles, potentially leading to sustained epidemics or chronic pathology. Understanding the interactions between nutrient enrichment and disease becomes increasingly crucial in tropical and subtropical regions, where nutrient inputs are predicted to rise in pathogen-rich environments, thereby necessitating disease management strategies to mitigate the intensification of global nutrient cycles [78].

## 3 Conclusions

**Fig. 2:**
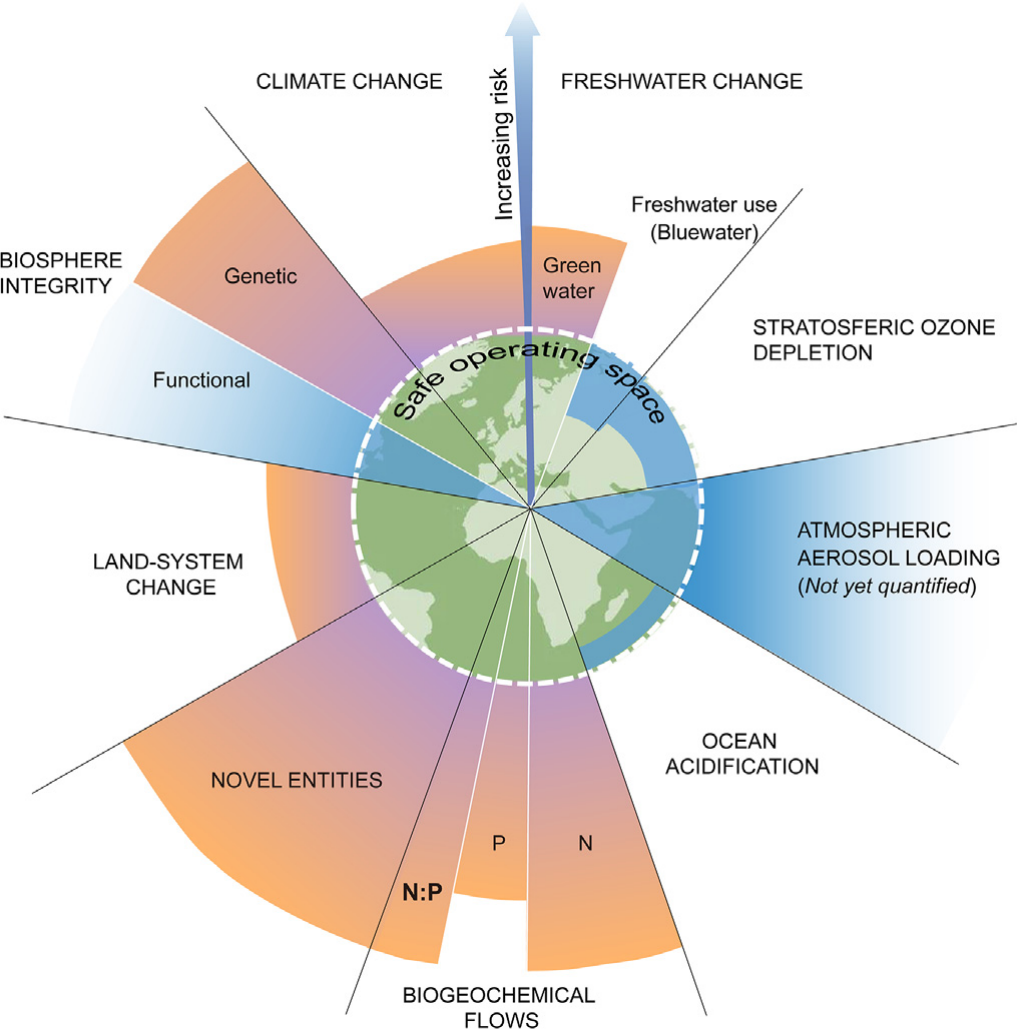
Planetary boundaries with additional consideration of the increasing N:P ratio.

Anthropogenic inputs of N and P have surpassed the planetary safety threshold, leading to severe consequences for ecosystems and global food security, but also very likely human health. Activities such as fertilizer use, pollution, and dietary changes have likely contributed to the increased prevalence of non-communicable diseases in developed countries, including lung and colon cancers, and coeliac disease. Additionally, in developing countries, these inputs have contributed to the spread of vector-borne diseases like malaria and Zika. However, several studies have also highlighted the potential benefits of controlling N, P, and N:P ratios in human intake as a therapeutic approach. This aligns with the observation of high concentrations of phosphorus-rich RNA, elevated P levels, and low N:P ratios in various neoplastic cells. Furthermore, recent research suggests that manipulating molecular N:P ratios can enhance the effectiveness of active molecular medicines, and improved management of N and P shifts holds promise for controlling vector-borne diseases. We advocate for deeper scientific and management consideration of anthropogenic nutrient imbalances in human health science under global change. Furthermore, we assert that the increasing N:P ratio and nutrient imbalances should be recognized as another of the planetary boundaries already crossed by humans (Fig. 2).

## Declaration of competing interest

The authors declare no competing interests at all.

## Author contributions

J.*P. and* J.S. designed the study, gathered the information and wrote the manuscript.
